# Evaluation of Susceptibility of *Aedes caspius* (Diptera: Culicidae) to Insecticides in a Potent Arboviral-Prone Area, Southern Iran

**DOI:** 10.18502/jad.v14i2.3748

**Published:** 2020-06-30

**Authors:** Sahar Hassandoust, Seyed Hassan Moosa-Kazemi, Hassan Vatandoost, Mohammad Mehdi Sedaghat, Kamran Akbarzadeh

**Affiliations:** 1 Department of Medical Entomology and Vector Control, School of Public Health, Tehran University of Medical Sciences, Tehran, Iran; 2 Department of Chemical Pollutants and Pesticide, Institute for Environmental Research, Tehran University of Medical Sciences, Tehran, Iran

**Keywords:** *Aedes caspius*, Susceptibility, Iran

## Abstract

**Background:**

Southern part of the country is a high risk for mosquito transmitted Arboviruses. This study was carried out to determine the base line susceptibility of the Aedini mosquitoes to the WHO-recommended insecticide.

**Methods:**

Larval collection was carried out by dipping method and adult collection occurred by suction tube from January to December 2017. The adult susceptibility test was assessed to Bendiocarb 0.1%, DDT 4%, Deltamethrin 0.05%, Lambda-cyhalothrin 0.05%, Malathion 5% and, Permethrin 0.75% at different interval times as well as at discriminative dose recommended by WHO. The larval susceptibility test was occurred using Temephos and *Bacillus thuringiensis* serotype H-14, at different concentrations. The LT_50_, LT_90_ and LC_50_, LC_90_ values were calculated for plotting the regression line using Microsoft office Excel software ver. 2007.

**Results:**

*Aedes caspius* was quite resistant to DDT, Malathion, Bendiocarb and showed susceptible or tolerant to other insecticides.The LT_50_ and LT_90_ values to DDT in this species were 157.896, and 301.006 minutes, respectively. The LC_50_ and LC_90_ values of *Ae. caspius* to Temephos were 0.000068, and 0.000130ppm, the figures for *B. thuringiensis* was 111.62 and 210.2ppm, respectively.

**Conclusion:**

A routine and continuous study for monitoring and evaluation of different species of *Aedes* to insectides is recommend at different parts of country for decision making.

## Introduction

Arthropod borne diseases are very important in the world. The tribe Aedini (Family Culicidae) contains approximately one-quarter of the known species of mosquitoes, including vectors of deadly or debilitating disease agents. This tribe contains the genus *Aedes*, which is one of the three most familiar genera of mosquitoes (1). The Aedini mosquitoes are responsible for transmission of the Barmah Forest, Batai, Babanki, Bouboui, Bunyamwera, Chikungunya, Cache, Valley, Dengue, Eastern Equine Encephalitis, Edge Hill, Everglades, Getah, GanGan, HighlandJ, Ilheus, James Canyon, Kedougou, La Crosse, Lebombo, Murray Valley River, Nyando, Ngari, Oriboca, Orungo, Pongola, Ross River, Rift Valley Fever, Semiliki Forest, Sindbis, St Louis, Encephalitis; Spondweri, Tahyna, Tensaw, Trivittatus, Uganda S, Venezuelan Equine Encephalitis, West Nile, WSLV, Wesselbron, Wyeomyia, Yellow Fever, and Zika (1). The number of Dengue cases reported annually by WHO ranged from 0.4 to 1.3 million in the decade 1996–2005 (2). As an infectious disease, the number of death cases varies substantially from year to year (3). At the present, Culicidae includes; 2 sub families, 11 tribes, 113 genera and 3526 species (4). The Iranian mosquitoes includes 69 species, that 7 or 11 genera depending on the classification used for aedines (5–6). Recent epidemics of mosquito-borne viral infections in countries neighboring Iran i.e. dengue, chikungunya and West Nile infections in Pakistan, dengue and Rift Valley fever in Saudi Arabia, and West Nile infection in Iraq have placed this country at a serious risk for mosquito-borne diseases (7–9). *Aedes caspius* (Pallas) is the vector of Tahina and West Nile Viruses (7–8, 10). At the present seven *Anopheles species* reported as the malaria vectors in the country including: *An. fluviatiliss*. *l*, *An. culicifaciess*. *l*, *An. sacharovi*, *An. maculipenniss*. *l*, *An. superpictus*, *An. stephensi* and *An. dthali* (11). In addition, Zaim et al. reported the *An. pulcherrimus*as secondary vectors of malaria in the South East of Iran (12). Oocyte of *Plasmodium* found at the first time in *An. multicolor*, while not found in salivary glands (13). Avian malaria reported in Iran by Ghaffari (14). Spraying with residual insecticide (IRS) considered an important mosquito control measure. Twelve insecticides recommended by WHO for IRS currently, which belong to four chemical groups including one organochlorine, six pyrethroids, three organophosphates and two carbamates (15–16). DDT resistance in the adult of *Aedes aegypti*, *Ae. albopictus* and susceptibility to Temephos, *Bacillus thuringiensis* and metabolic resistance of the current species to deltamethrin and DDT have been reported in Africa (17). Resistance of *Ae. aegypti* larvae to Temephos has been reported in Asia (18–19). In addition, larval resistance of *Aedes albopictus* to Temephos have been reported in Malaysia (20), Thailand (21).Adult susceptibility test on *Ae. aegypti* against some payrethroids has been reported in various research study (21–24). In spite of some reports due to resistance of *An. stephensi* against DDT, Dieldrin and Malathion in Iran (13, 25–31). Mechanism of resistance of *An. stephensi* against temephos has been reported by (32–33). By now there are no evidence of resistance of *Ae. vexans* and *Ae. caspius* in Iran. Release of larvivorous fish and microbial agent using the *Bacillus thuringiensis*, and larviciding by chlorpyrifos-methyl are the main larval control measures and pyrethroid as new insecticides are being used as IRS and LLINs in Iran (34–35). In spite of more than 50 years’ malaria control programming more than 60% of the total malaria cases reported from Southern Iran. Malaria is one of the most important communicable diseases transmitted by anopheline mosquitoes (Diptera: Culicidae) to humans. In 2013, there are 97 countries and territories with ongoing malaria transmission, and 7 countries in the prevention of reintroduction phase, making a total of 104 countries and territories in which malaria is presently considered endemic. Based on WHO estimate, 207 million cases of malaria occurred globally in 2012 resulted to 627 000 deaths (2). Malaria is one of the important infectious diseases in Iran with an average of about 15000 annual cases in the last decade, while total recorded cases has dropped to less than 500 locally transmitted cases in 2013. More than 80% of malaria cases in Iran are reported from three provinces of Sistan and Baluchistan, Hormozgan and Kerman in southern and southeastern areas of the country. The most routes of malaria cases are immigration from Afghanistan and Pakistan to southern and southeastern areas of the country (36). Over the last 20 years there has been a dramatic reduction of the malaria burden in Iran. While in 1991, nearly 100,000 cases were reported, less than 100 locally transmitted cases in 2017 (Ministry of Health, annual reports unpublished data). All observations indicate that the data reflect the real situation and that the overwhelming majority of cases, which occur, are included in the national system, although there is room for improvement in the surveillance system. The spectacular progress can be ascribed to effective implementation of appropriate curative and preventive control interventions through a strong health care infrastructure. Social and economic development allowing better housing, use of air-conditioning etc. has also played a role. Locally transmitted cases are now concentrated in the southeastern part of the country, which are affected by extensive population movement across the border with Pakistan, where malaria control faces serious difficulties. In 2009, Iran set time-bound elimination objectives for its malaria program. There has been excellent progress since, but the continued risk of importation of malaria cases from Pakistan poses a huge challenge, politically, socially, operationally and technically, to malaria elimination in Iran. The situation in the next decade will be absolute elimination or one where a few small short-lived foci emerge from time to time as a result of importation. The latest number of autochthonous cases in the whole country is 42 including 23 local malaria patients, 7 relapsed cases, 12 imported from the other districts by end of July 2016 (Ministry of Health, annual reports unpublished data). *Aedes albopictus* and *Ae. aegypti* has been recently reported in Algeria, Lebanon, Palestine, Syria, and Turkey (37). *Aedes albopictus* has been identified along the south eastern Iran (6) and Mediterranean coast of Europe for decades along with local transmission of DENV and chikungunya since 2007 (38). Near the Pakistan border, serologic evidence suggests possible DENV transmission in Iran (39–41), in Afghanistan (42), though local transmission has not been confirmed to our knowledge (41). The presence of *Aedes* or DENV transmission in these areas should not be ruled out (41). Qeshm and Kish are commercial and industrial free zones in Hormozghan Province. This area also is important due to agricultural and husbandry in southern Iran in the border line of Persian Gulf and Oman sea. The study area is endemic to malaria, however in recent years, the nuisance’s aedini species have been increased. There are no data about susceptibility level of Aedini vectors in Iran, so, the susceptibility level of Aedeini mosquitoes has been studied during this research. The results could provide an essential clue for judicious use of insecticides and will be very useful to health authorities for future planning of vector control.

## Material and Methods

### Study area

**Fig. 1.**
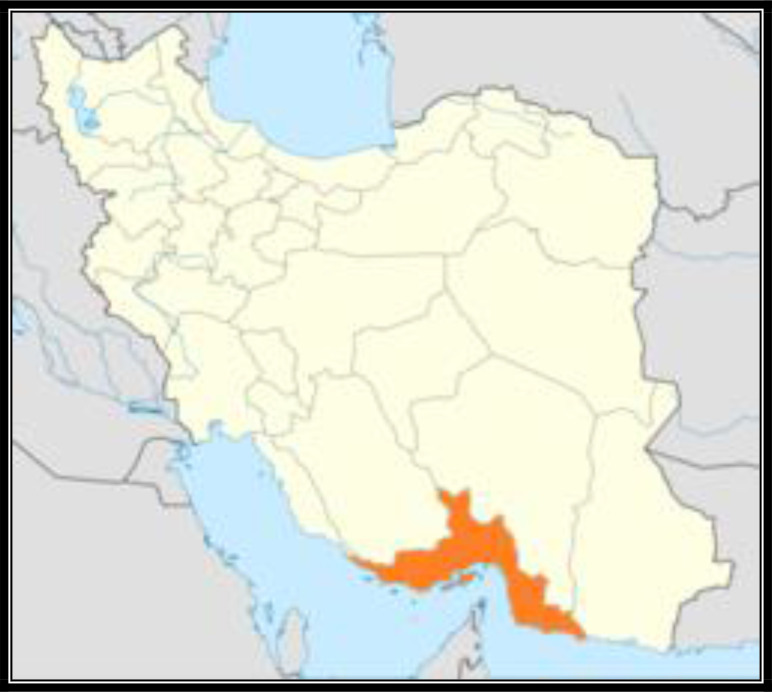
Map of study area, Hormozghan province, Iran

The study was carried out in Hormozgan (27°11′18″N 56°16′36″E/27.1884°N 56.276 8°E), Province, southern Iran. The people engaged to agriculture, horticulture, livestock, fishing sailing, and hand crafts including needlework, making carpet and musical instruments. The absolute maximum and average of temperature was reported 52 °C and 26.5 °C in Hormozghan Province, respectively. Average annual rainfall and humidity was 140.28mm and 79%, respectively. The absolute maximum and average of temperature in Isfahan was reported 40.6 °C and 17.1 °C. In this area average annual rainfall and humidity was 63.5 mm and 22%, respectively (43) (Fig. 1).

Hormozghan province with 70,697km^2^ (27,296 sq. mi) square kilometers comprised of 21 counties (or districts), 69 municipalities, 13 major cities and 2,046 villages. In 2011 a little more than 1.5 million people resided in Hormozghan Province. Daregaz village (27° 49′GN, 56°17′GE) with 268 households, and 926 populations, and Kovae village (27°44′GN, 56°22′GE), 38 households and 112 populations, Talsooro village (27°46′GN, 56°23′GE), 92 households and 309 populations, as fixed stations and Zakin village (27°49′GN, 56°16′GE), 158 households 571 population selected randomly as variable stations.

### Sampling methods

Sampling methods such as larval collection, hand catch was carried out during January to December 2017 (44). These studies were conducted once every 30 days and collected mosquitoes were identified by specific systematic keys (5, 45).

### Larval collection and rearing

In each fixed and variable station larvae was collected from January to December 2017. Mosquitoes larvae were picked up from the water using a dropper, pipette or fine net and inserted into the bulb. The related data such as water temperature, larval type, number and date sampling was recorded. Larvae and pupae in holding container filled with water were transferred to the laboratory for rearing. Mosquito larvae feed by dry fish food. Adult mosquitoes live quite well on bowl of sucrose 5% in bottom of the cage. The adults were kept at 28 °C, relative humidity (80%) and 14L-10D photoperiod (46).

### Hand collection

Aedini mosquitoes were collected from the villages between 06.30 and 09.30AM. Sampling was carried out in each human dwelling, cattle and goat sheds for 15min using suction tube and torch (44). The mosquitoes were transferred in the cage as dimensions of 40×40cm and then sent to the laboratory. Total of 200 to 250 mosquitoes were entered in each cage and covered with wet towel. The sucrose 5% solution was placed inside the cage. The mosquitoes were kept in standard condition (25 °C, 75% RH). In Hormozghan Province, totally, ten species were collected including: *An. stephensi*, *An. dthali*, *An. culicifacies*, *An. fluviatile*, *Cx. pipiens*, *Cx. quinquefasciatus*, *Cx. theileri*, *Culiseta longiareolata*, *Ae. caspius*s. l and *Ae. vexans*. In adult collection *An. stephensi* was dominant species 34.76% allocated mosquitoes collected. *An. dthali* and *An. culicifacies* were followed 15%, 12.92%, respectively. *Culiseta longiareolata* had the lowest density with 1.09%. *An. culicifacies*, *An. stephensi*, *Cx. pipiens*, *Cx. theileri* were collected in all months. In larval collection, *An. stephensi*, with 1495 specimens (28.9%) was predominant followed by *Cx. pipiens* 753 (14.1%), *An. culicifacies*12.8%, *Cx. quinquefasciatus* 6.3% in the same month. It should be noted that *Aedes caspius* larvae was collected in May and December.

### Insecticide impregnated papers

Impregnated papers with DDT 4%, malathion0.08%, bendiocarb 0.1%, deltamethrin 0.05%, lambda-cyhalothrin 0.03%, permethrin 0.25%, and control papers were supplied by World Health Organization.

### Larvicides solutions.

Five concentration of Temephos as (0.000015, 0.000031, 0.000062, 0.000125, 0.000250ppm) and four concentrations of Bti as (4, 36, 296, 2368ppm) were immersed in 249mL of tap water separately and larval test was applied based of WHO criteria guideline 2016 (2).

### Adult susceptibility test

The adult susceptibility test was carried out according WHO guideline (2). Each time 4–5 mosquito collected and insert to holding tube overall 20–25 mosquito were kept into holding tube. The susceptibility tests performed on their standard condition (22–26 °C, 60% H). The susceptibility of the wild strain of Aedini mosquitoes was assessed to the insecticides impregnated papers. The mosquitoes were exposed to different insecticides by different interval times and 24 hours’ recovery period.

### WHO criteria for susceptibility test

Based on WHO recommendations (2), the following criteria have been used for interpretation and classification; Mortality in the range 98–100% indicates susceptibility. A mortality of less than 98% is suggestive of the existence of resistance and further investigation is needed. The observed mortality (corrected if necessary) is between 90% and 97 %, the presence of resistant genes in the vector population must be confirmed. The confirmation of resistance may be obtained by performing additional bioassay tests with the same insecticide on the same population or on the progeny of any surviving mosquitoes (reared under insectary conditions) and/or by conducting molecular assays for known resistance mechanisms. If at least two additional tests consistently show mortality below 98%, then resistance is confirmed. If mortality is less than 90%, confirmation of the existence of resistant genes in the test population with additional bioassays may not be necessary, as long as a minimum of 100 mosquitoes of each species was tested. However, further investigation of the mechanisms and distribution of resistance should be undertaken. When resistance is confirmed, pre-emptive action must be taken to manage insecticide resistance and to ensure that the effectiveness of insecticides used for malaria vector control (2).

### Identification of mosquitoes using morphological Characteristics

The mosquitoes after the test were mounted and identified by specific systematic keys. The samples were recorded in the special forms by and the appropriate time of deaths Associated with history of collection, relative humidity and temperature (5, 45).

### Statistical analysis

Results were considered reliable if the control mortality was less than 5% and rejected if more than 20%. Results were corrected by Abbott’s formula when mortality rates of control group were between 5 to 20% (47–48). Data were analyzed by probit analysis (49). Regression lines of the species were measured through the χ2 test. The LT_50_ and LT_90_values were calculated for plotting the regression line using Microsoft Excel software ver. 2013.

## Results

### Adult bioassay

**Fig. 2.**
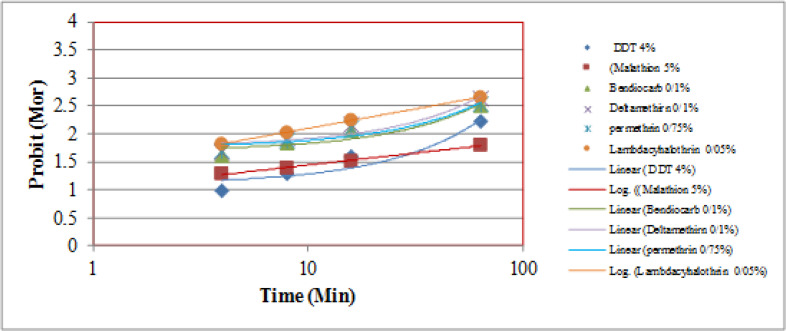
Regression line of *Aedes caspius* Adult stage exposed to Some Insecticides Recommended by WHO in arboviral-prone Area Southern Iran, 2015

Adult bioassays using various insecticides showed that LT_50_ and LT_90_ values for DDT 4% against *Ae*. *caspius* were ranged from 157.896–301.006 minutes for the BAND strain. Bioassay test for other insecticides against is shown in Table. 1, Fig. 2.

**Table 1.** Regression line parameters of *Aedes caspius* adult stage exposed to some insecticides recommended by WHO in a arboviral-prone Area Southern Iran, 2017

**Insecticide**	**A**	**B±SE**	**LT**_**50**_ **95% C.I (minute)**	**LT**_**90**_ **95% C.I (minute)**	**χ**^**2**^**(df)**	**P-Value**	**book**	**Y= BX+A**
**DDT4%**	1.1511	0.0167±0.196	157.896	301.006	2.925 (2)	P>0.05	5.99	y= 0.0167x+1.1511
**Malathion 5%**	1.2944	0.0081±0.190	160.229	304.435	0.289 (2)	P>0.05	5.99	y= 0.0081x+1.2944
**Bendiocarb 0.1%**	1.6845	0.0135±0.087	42.124	80.0356	0.357 (2)	P>0.05	5.99	y= 0.0135x+1.6845
**Deltamethrin 0.1%**	1.7745	0.0141±0.077	48.735	92.5965	0.08 (2)	P>0.05	5.99	y= 0.0141x+1.7745
**Lambda-cyhalothrin 0.05%**	1.8494	0.0132±0.166	46.129	87.6451	11.307 (2)	P>0.05		y= 0.0132x+1.8494
**Permethrin 0.75%**	1.5955	0.0156±0.196	29.652	56.3388	10.890 (2)	P<0.05	5.99	y= 0.0156x+1.5955

### Larval bioassay

**Fig. 3.**
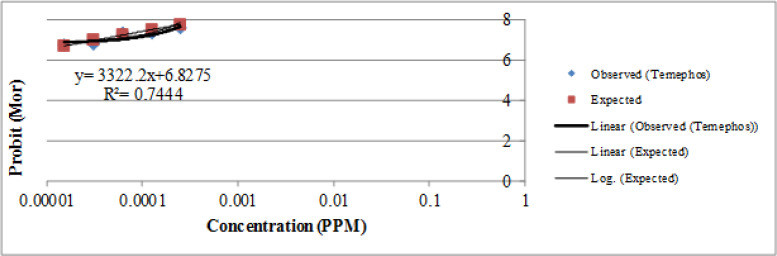
Regression line of *Aedes caspius* larval stage exposed to Temephos Larvicide Recommended by WHO in a potent Dengue Endemic Area of Central and Southern Iran, 2015

**Fig. 4.**
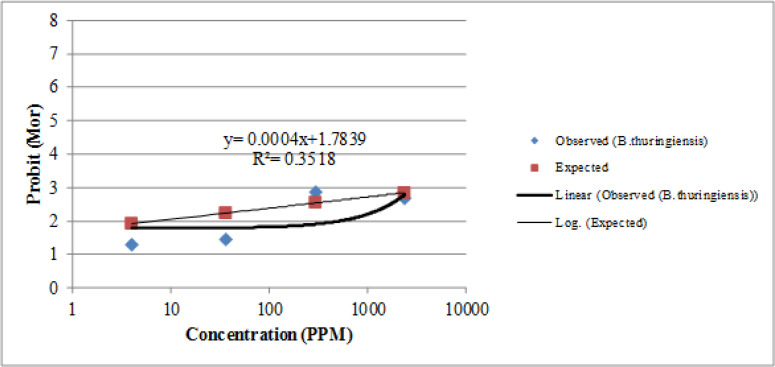
Regression line of *Aedes caspius* Larval stage exposed to *B. thuringiensis* Larvicides Recommended by WHO in arboviral-prone Area Southern Iran, 2015

Larval bioassays using Temephos showed that LC_50_ and LC_90_ for *Ae. caspius* ranged from 0.000068–0.000130mg/l for the BAND strain (susceptible reference strain) to 111.62–210.2 mg/L for the B. T (Table. 2, Figs. 3, 4).

**Table 2.** Regression line parameters of *Aedes caspius* larval stage exposed to Some Larvicides Recommended by WHO in arboviral-prone Area Southern Iran, 2017

**Larvicide**	**A**	**B±SE**	**LC**_**50**_ **95% C.I**	**LC**_**90**_ **95% C.I**	**χ**^**2**^	**P-Value**	**book**	**Y= BX+A**
**Temephos**	6.8275	3322.2±0.385	0.000068	0.000130	0.872 (3)	P>0.05	7.81	y= 3322.2x+ 6.8275
***B.thuringiensis***	1.7839	0.0004±0.256	111.62	210.2	173.914 (2)	P<0.05	7.81	y= 0.0004x+1.7839

Mortality of *Aedes caspius* exposed to DDT and other insecticides has shown in Tables 1 and 2. LT_50_ and LT_90_ values of this species to DDT 4% were 157.89 and 301.006 minutes, respectively. This species was quite resistant to DDT and other insecticides except deltamethrin (Fig. 2).

It is concluded that *An. caspius* is resistant to DDT, malathion, and bendiocarb, permethrin, lambdacyhalothrin whereas susceptible to deltamethrin, (Table 1). The LT_50_ and LT_90_ values of this species to DDT 4% were 157.896 and 301.006 minutes (Table 1, Fig. 2).

Mortality of *Aedes caspius* larvae exposed to temephos and *Bti* has shown in (Table 2 and Figs. 3, 4). LC_50_ and LC_90_ values of this species to temephos were 0.000068 and 0.000130ppm, respectively. LC_50_ and LC_90_ values of this species to *Bti* were 111.62 and 210.2ppm, respectively.

## Discussion

In our study, 4 genera and 10 species of mosquito larvae and adults were identified based on morphological characters. Culicidae species were belongs to the genus of *Anopheles*, *Culex*, *Culisita*and *Aedes*. The species of *Ae. caspius* and *Ae. vexans* found by larval collection. The most predominant species was *An. stephensi* with 34.76% of adult and 29.36 % of larvae collection. Vatandoost et al. (2004b) (50), reported three biological forms of this species including type, intermediate and mysorensis in southern Iran. Type and intermediate forms cited as vector in urban areas whereas, mysorensis form as vector in rural area (51). In Iran, indoor residual spraying (IRS) with DDT was carried out for malaria control during 1950–1968. In this species, resistance to DDT was first recognized in 1958 malathion in 1976 (13). Following the emergence of resistance of *An. stephensi* to DDT, other organophosphorus, carbamate and pyrethroid insecticides were used. The susceptibility level of *An. stephensi* to DDT and Dieldrin was studied at various parts of Iran bordered in Persian Gulf and Oman Sea during 1985–2016. The situation of Dengue fever and dengue hemorrhagic fever has been changed in imported to indigenous cases in Iran and probable *Aedes albopictus* is responsible for these endemic diseases due to unplanned urbanization (6). In southern Iran, the climatic conditions are suitable for mosquito’s life cycle. The changes in temperature, humidity and wide range of water grades may have a significant effect on the population growth and also vector control programmers (52). Potent dengue vector in Iran has exophilic behavior, so, the efficacy of larvicing materials is very important to vector control programs. Temephos and *Bti* were evaluated in Lab scale against *Ae. caspius* larvae in the current study. In this research work, different concentrations of *Bti* were prepared as done by previous workers (53–54). *Bacillus turingiensis* is safe and effective biocontrol agent used widely to control of mosquitoes for the recent years (55–58). The experiment was conducted in tape water. Abdalmagid et al. (2012) (53) checked the efficacy of *Bti* dunks in field water and studied the physio-chemical properties of water. They concluded that these properties have no impact on the efficacy of Bti (P> 0.05). Mulla (1990) (59) studied that it was difficult to handle 1^st^ instar larvae because of high mortality rate during handling. Due to this reason we used 3^rd^ and 4^th^ instar larvae for our experiments. In the present study we found low mortality rate in case of *Bti*. In agreement with this study, Rodrigues et al. (1999) (60) reported the low mortality of *Ae. agypti* post treatment by *Bti* and 24h. Recovery periods. Ramathilaga et al. (2012) (61) studied the impact of *Bti* against 3rd instar larvae of *Ae. aegypti* as was recorded in the present study against 3^rd^ and 4^th^ instar larvae of *Ae. caspius*. In the present study, 40% and 78% mortality was recorded for 592 and 1184ppm of *Bti* respectively after 24h in tape water while Ramathilaga et al. (2012) (61) recorded (16%) mortality at the 1mg concentration of *Bti* for 24h treatment in tap water. Haung et al. (1993) (62) recorded 52.1, 69.5 and 78.2% mortality after 12, 24 and 48h respectively in 0.10ppm against *Ae. aegypti* larvae while 97.1, 97.1 and 97.1% mortality after 12, 24 and 48h in 0.20ppm. Gbehou et al. (2010) (63) compared the efficacy of *Bti* on *Aedes*, *Culex* and *Anopheles* species and observed 40, 80 and 100% mortality after 2, 4 and 6h against *Aedes* species. Many other factors such as species, genera susceptibility, feeding behavior of larvae, instar susceptibility to biocides, suspended organic matter, water temperature, larval density, and water depth influence the efficacy of *Bti* against mosquitoes (Boisvert 2005) (64). Some of these factors like organic, inorganic, muddy, food and floating particles decreased the efficacy of Bti due to adsorption of *Bti* onto suspended particles followed by a slow sedimentation (65–66). In the present study, we found higher concentration of *Bti* is enquired for 100% mortality rate. In parallel, Ohana et al. 1987 (67), Mulla 1990 (59) reported more concentration need to control of *Ae. agypti* larvae due to *Bti* a few toxic suspended crystals particles ingested by larvae. In this research study, different concentrations of Temephos were prepared as done by previous workers (68). This larvicides is safe and effective agent used widely to control of mosquitoes for the recent years. Kemabonta and Nwankwo 2013 (68) checked the efficacy of Temephos in field water with comparison to spinozad. They concluded that these properties have good impact on the 3^rd^ and 4^th^ *Ae. agypti* larvae (P> 0.05). The LC_50_ values for wild *Aedes caspius* larvae were 0.000068mg/l and 0.000130mg/l, while the LC_50_ values for the laboratory bred and wild *Aedes aegypti* larvae were 7.418g/l and 8.150 g/l respectively (68). In the present study, 100% mortality was recorded at 0.000250mg/L of temephos respectively after 24h in tape water while Kemabonta and Nwankwo (2013) (68) recorded (100%) mortality at the 30g/L concentration of temephos for 24h treatment in tap water. Many other factors such as species, genera susceptibility, feeding behavior of larvae, instar susceptibility to biocides, suspended organic matter, water temperature, larval density, and water depth influence the efficacy of *Bti* against mosquitoes (64). Some of these Many other factors like organic, inorganic, muddy, food and floating particles decreased the efficacy of Temephos. In addition, many factors effects of efficacy of Bti due to adsorption of Bti onto suspended particles followed by a slow sedimentation (65–66).

In the present study, we found higher concentration of *Bti* will be needed for 100% mortality rate. In parallel, Ohana et al. 1987 (67), Mulla 1990 (59) were reported more concentration need to control of *Ae. agypti* larvae due to *Bti* a few toxic suspended crystals particles ingested by larvae.

The interruption in the efficacy of *Bti* was found to be caused by bacterial adsorption to soil particles, but the inactivation could be inverted by washing the mud away (44). Due to these reasons, the mean value of LC_50_ was higher against *Ae. caspius* larvae in comparison to temephos. The mean LC_50_ values of *Bti* and Temephos were 111.62ppm and 0.000068ppm after 24h for tape water respectively. The results of the present study revealed the higher mortality post treatment by Temephos in tape water because temephos is considered as contact larviciding in comparison to *Bti* as digestive effects and it is free of any particles due to suspended particles. Based on the literature, no reports were available on the susceptibility levels of *Ae. caspius*.

## Conclusion

Iran is near the Dengue endemic area, *Aedes albopictus* was reported for the first time in southeastern Iran in 2014. By now, IRS in human dwelling sand animal shelters, space-spraying, personal protection through distribution of LLINs and curtains (ICNs), repellents measures used to control of vectors in Iran. In addition, some biological and chemical agents against larval and adult stages of mosquitoes had been evaluated in the laboratory. Results obtained from susceptibility tests of *Ae. caspius* on some WHO recommended insecticides revealed that highly resistance to them in southern Iran. Precautionary measures should be taken in future vector control operations. Moreover, the status of resistance in other locations in this area should be investigated. Since the country relies on deltamethrin for IRS operation, tolerant populations of *Aedini* species implies careful consideration and regular monitoring of susceptibility level of mosquitoes in the future.
